# Emissions of CH_4_ and N_2_O under Different Tillage Systems from Double-Cropped Paddy Fields in Southern China

**DOI:** 10.1371/journal.pone.0065277

**Published:** 2013-06-04

**Authors:** Hai-Lin Zhang, Xiao-Lin Bai, Jian-Fu Xue, Zhong-Du Chen, Hai-Ming Tang, Fu Chen

**Affiliations:** 1 College of Agronomy and Biotechnology, China Agricultural University, Key Laboratory of Farming System, Ministry of Agriculture, Beijing, China; 2 Patent Examination Cooperation Center of the Patent Office, SIPO, Beijing, China; 3 Soil and Fertilizer Institute of Hunan Province, Changsha, China; DOE Pacific Northwest National Laboratory, United States of America

## Abstract

Understanding greenhouse gases (GHG) emissions is becoming increasingly important with the climate change. Most previous studies have focused on the assessment of soil organic carbon (SOC) sequestration potential and GHG emissions from agriculture. However, specific experiments assessing tillage impacts on GHG emission from double-cropped paddy fields in Southern China are relatively scarce. Therefore, the objective of this study was to assess the effects of tillage systems on methane (CH_4_) and nitrous oxide (N_2_O) emission in a double rice (*Oryza sativa* L.) cropping system. The experiment was established in 2005 in Hunan Province, China. Three tillage treatments were laid out in a randomized complete block design: conventional tillage (CT), rotary tillage (RT) and no-till (NT). Fluxes of CH_4_ from different tillage treatments followed a similar trend during the two years, with a single peak emission for the early rice season and a double peak emission for the late rice season. Compared with other treatments, NT significantly reduced CH_4_ emission among the rice growing seasons (*P<0.05*). However, much higher variations in N_2_O emission were observed across the rice growing seasons due to the vulnerability of N_2_O to external influences. The amount of CH_4_ emission in paddy fields was much higher relative to N_2_O emission. Conversion of CT to NT significantly reduced the cumulative CH_4_ emission for both rice seasons compared with other treatments (*P*<0.05). The mean value of global warming potentials (GWPs) of CH_4_ and N_2_O emissions over 100 years was in the order of NT<RT<CT, which indicated NT was significantly lower than both CT and RT (*P*<0.05). This suggests that adoption of NT would be beneficial for GHG mitigation and could be a good option for carbon-smart agriculture in double rice cropped regions.

## Introduction

With the current rise in global temperatures, numerous studies have focused on greenhouse gases (GHG) emissions [1]–[3]. Agriculture production is an important source of GHG [4]. In addition to carbon dioxide (CO_2_), methane (CH_4_) and nitrous oxide (N_2_O) also play an important role in global warming. The global warming potentials (GWPs) of CH_4_ and N_2_O are 25 and 298 times that of CO_2_ in a time horizon of 100 years, respectively [5]. In addition to industrial emissions, farmland is another important source of atmospheric GHG [6]–[9]. Numerous results indicate rice (*Oryza sativa* L.) paddy field is a significant source of CH_4_ [9], [10]. The anaerobic conditions in wetland rice field are favorable for fostering CH_4_ emission [11].

A considerable number of studies have shown that some farm operations can influence CH_4_ and N_2_O emission. For example, water/nitrogen (N) management, organic matter application and tillage can regulate CH_4_ and N_2_O emission [12]–[14]. Tillage and crop residues retention have a great influence on CH_4_ and N_2_O emission through the changes of soil properties (e.g., soil porosity, soil temperature and soil moisture, etc.) [15], [16]. In some experiments, conversion of conventional tillage (CT) to no-till (NT) can significantly reduce CH_4_ and N_2_O emission [17], [18]. However, tillage effects on CH_4_ and N_2_O emission are not always consistent among different studies. Dendooven et al. reported that CH_4_ emission were not significantly affected by tillage [19]. In addition, some studies show that crop residues retention can increase CH_4_ and N_2_O emission from paddy fields [20]–[22].

Most previous studies of CH_4_ and N_2_O emissions in paddy field have focused on the effects of water and N management on GHG emission [23]–[26]. However, tillage can result in changes to GHG emission through the alteration of soil properties and biochemical processes. Although CT is widely adopted around the world, it strongly disturbs the soil, consumes more energy, and even leads to disaster (i.e., the 1930s Dust Bowl in the U.S.). Conservation tillage is increasingly being adopted in the world because of the numerous benefits (e.g., saving time/energy/fuel, controlling soil erosion and increasing water use efficiency). Presently, more and more countries in Asia are facing the problem of labor shortages and high labor cost in planting rice. Conservation tillage in paddy fields (e.g., NT, direct seeding) has increasingly been adopted in Asia, especially in Southern China. Currently, the labor shortage in agriculture has been a major constraint confronting rural China. Because of energy and labor savings, NT has been widely adopted as a principal conservation technology in China. Furthermore, it is estimated that about 2.18×10^8^ Mg yr^−1^ of rice crop residues are generated in China, accounting for 27.51% of the gross crop residue production [27]. Xiao et al. [28] reported that only 9.81% of crop residue was returned to croplands as fertilizer, but >20% of crop residue was burned directly in the field or thrown away, thus increasing environmental pollution and threatening public safety. Therefore, rational use of tillage and crop residues is of great importance for GHG emission mitigation in China.

Until now, most studies on GHG emissions in paddy fields have been based on single rice (one rice cropping in one year) or rice–wheat (*Triticum aestivum* L.) cropped fields and very few studies have involved tillage impacts on emissions of CH_4_ and N_2_O in double rice (two rice crops in one year, early rice and late rice) cropped fields [4], [12], [29]. The lower Yangtze region is a typical double rice cropped area in China, accounting for 40–60% of total arable land in this region [30]. Due to the important role of rice paddies in global agriculture, adopting reasonable agricultural management is of great importance in the mitigation of global GHG emissions. Therefore, it is valuable to examine GHG emissions in paddy fields under different tillage systems and to improve reasonable practices for mitigation of GHG emissions. The objective of this paper was to assess tillage effects on emissions of CH_4_ and N_2_O, and to identify the influencing factors controlling CH_4_ and N_2_O emission under different tillage methods.

## Materials and Methods

### Ethics Statement

This experiment was established in a long-term experiment site (Ningxiang, 112°18′E, 28°07′N, Hunan province, China), which belongs to Soil and Fertilizer Institute of Hunan Province. This research was performed in cooperation with China Agricultural University and Soil and Fertilizer Institute of Hunan Province. The farm operations of this experiment were similar to rural farmers’ operations and did not involve endangered or protected species. The experiment was approved by the Key Laboratory of Farming System, China Agricultural University and Soil and Fertilizer Institute of Hunan Province.

### Site Description

The experimental area has a subtropical monsoonal humid climate, with an annual average precipitation of 1358.3 mm and annual average temperature of 16.8°C. The typical cropping system in this area is double rice cropping in a year (i.e., early rice and late rice). Normally, rotary tillage is conducted one or two days before rice seedling transplanting. Principal properties of the surface soil (0–20 cm) are presented in Table 1. The experimental site had been cultivated with rice under rotary tillage (RT) without crop residue retention for ∼30 years before the initiation of the experiment. Generally, early rice is transplanted in early April and harvested in early July and late rice is immediately transplanted after the early rice harvest and is subsequently harvested in middle October.

**Table 1:** Principal soil properties of the test soil.

Soil layer (cm)	Bulk density(g cm^−3^)	Soil organic matter(g kg^−1^)	Available N(mg kg^−1^)	Available P(mg kg^−1^)	Available K(mg kg^−1^)	pH (H_2_O)
0–20	1.21	34.90	224.10	4.38	97.10	6.26

### Experimental Design and Treatments

The field experiment was established in 2005 with three tillage treatments: conventional tillage (CT), rotary tillage (RT) and no-till (NT). The treatments were laid out in a randomized complete block design with three replications and the area of each plot was 66.7 m^2^. For all treatments, rice residue was retained on the soil surface after rice harvest until tillage operations were conducted. No-till operation was conducted in NT and the rice residue was retained on the soil surface throughout the entire study period. The CT plots were plowed once to a depth of ∼15 cm using a moldboard plow and rotavated twice to a depth of ∼8 cm on the day of rice seedling transplanting. The RT plots were rotavated four times to a depth of ∼8 cm on the day of rice seedling transplanting.

Early rice (*Zhongjiazao 32#*) was transplanted on April 7, 2007 and April 10, 2008. Late rice (*Xiangwanshan 13#*) was transplanted on July 10 both in 2007 and 2008. All plots received 375 kg ha^−1^ compound fertilizer(N:P_2_O_5_:K_2_O = 20∶12∶14)as basal fertilizer at seedling transplanting. One week after seedling transplanting, the plots were top-dressed with urea (46% of N), 150 kg ha^−1^ for the early rice and 75 kg ha^−1^ for the late rice. Selective herbicides (34% Quinclorac, 4% Bensulfuron-methyl) were applied prior to rice transplanting in all treatments. The planting density was ∼803 640 strains ha^−1^ and ∼12 500 kg ha^−1 ^yr^−1^ of rice residue was retained to the soil during the experimental years.

### Data Collection

Soil temperature was measured by thermometers (DF-201A, Beijing Dongfang Mingguang Electronic Science And Technology Co., Ltd) with a measuring range of −30°C to +100°C. The thermometers were inserted into the 5 cm and 10 cm soil depth and data were recorded at 10-day intervals after rice seedling transplanting. Soil bulk densities (ρ_b_) at 0–5 cm, 5–10 cm and 10–20 cm depth were determined by the core method.

Soil porosity (SP, m^3^ m^−3^) was calculated by using the formula below:
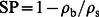
(1)Where, ρ_s_ is soil particle density, Mg m^−3^.

Soil samples were collected from each treatment plot prior to rice seedling transplanting and at the rice harvest.

Fluxes of CH_4_ and N_2_O were measured with the closed chamber method [31]. For each plot, three chamber bases were inserted into the soil (5 cm depth) after tillage operations. To avoid soil disturbance, every chamber base was placed at a fixed position until rice harvest. A removable wooden bridge (2 m long and 0.5 m wide) was placed near the chamber base for convenience of sampling. The chamber base had a 5 cm deep groove for installation. A chamber made with polymethyl methacrylate was placed at the chamber base. The cross-sectional area of each chamber was 0.36 m^2^ (0.6 m×0.6 m) and the height was 0.8 m. Chambers were closed by filling the groove of the base with water during gas sampling, and the chamber was equipped with a small fan to mix air inside the chamber. Gas samples were collected with vacuum vials. In order to minimize the underestimation of gas fluxes with the closed chamber method, the time-course of each gas sampling was kept within 10 min [32]. Measurements were conducted every 4 hours on each sampling day. Gas samples were collected at least three times per month. During the tillage period (∼1 week) and the field drainage period (∼10 days), gas collection was conducted daily. The gas samples were analyzed for CH_4_ and N_2_O using a gas chromatography with FID and ECD (model 6890N, Agilent Technologies, CA).

The fluxes of CH_4_ and N_2_O emissions were calculated by using the formula below [33]:
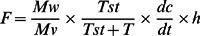
(2)Where *F* is the emission fluxes (mg m^−2^ min^−1^); *M_w_* is the molar mass of trace gas (g mol^−1^); Mv is the molar volume of trace gas (L mol^−1^); *T_st_* is the absolute temperature (273.2 K); *T* is the air temperature at sampling (°C); *dc*/*dt* is the change in the rate of CO_2_ or CH_4_ concentrations (ppbv min^−1^); and *h* is the height of the chamber (m).

The cumulative emissions within one year were calculated assuming the existence of linear changes in gas fluxes between two successive sampling dates. Meteorological data were obtained from China National Meteorological Bureau.

GWPs is defined as the cumulative radiative forcing both direct and indirect effects integrated over a period of time from the emission of a unit mass of gas relative to some reference gas [34]. Carbon dioxide was chosen as this reference gas. The GWPs conversion parameters of CH_4_ and N_2_O (over 100 years) were adopted with 25 and 298 kg ha^−1^ CO_2_-equivalent [35].

### Statistical Analyses

Statistical analyses were performed with SPSS 11.0 analytical software package (SPSS Inc., Chicago, IL, US). Statistical analysis was performed with ANOVA to analyze the effects of tillage on ρ_b_, SP, CH_4_ and N_2_O flux among the treatments. The Tukey-HSD was calculated for comparison of the treatment means. With regard to CH_4_ and N_2_O fluxes, data for each sampling day were analyzed separately. Differences among treatments were declared to be significant at *P<0.05*.

## Results

### Air Temperature and Precipitation

In general, air temperature during May and September ranges from 22 to 30°C in this region. April and October are the coldest months during the rice growing period, with mean air temperature ∼20°C. The mean air temperature in 2007 was higher than that of other years, but the air temperatures were slightly lower than the average of other years in September and October of 2007 (Table 2). Mean precipitation changed dramatically compared with the two years, 81.4 mm in 2007 and 32.8 mm in 2008. The precipitation is mainly distributed between May and August, especially during May and June in this region. However, the precipitation in August and September of 2007 was more than the average and these months had the highest precipitation in 2007. Precipitation in 2008 was much less compared to that of other years (Table 2).

**Table 2:** Mean monthly precipitation and air temperature from April to October between 2005 and 2008 at the experimental site.

Month	Precipitation (mm)				Air temperature (°C)							
	2005	2006	2007	2008	2005	2006		2007		2008		
April	92.2	235.0	38.0	26.3	20.6		19.9		25.8		18.7	
May	400.8	125.0	119.0	27.3	22.6		23.6		26.6		24.5	
June	272.1	201.0	119.0	25.6	27.2		27.0		26.6		26.6	
July	66.7	133.0	44.0	30.9	30.2		30.1		30.8		30.0	
August	80.4	154.0	126.0	58.1	27.0		29.5		29.6		28.7	
September	47.5	18.0	121.0	43.2	24.6		24.0		23.5		25.6	
October	64.4	40.0	3.0	18.2	18.2		21.3		19.4		20.2	
Mean	146.3	129.4	81.4	32.8	24.3		25.1		26.0		24.9	

Source: China Meteorological Data Sharing Service System. These data represent the mean monthly precipitation and temperature. The early and late rice growing period was April to October.

### Soil Bulk Density

**Figure 1:**
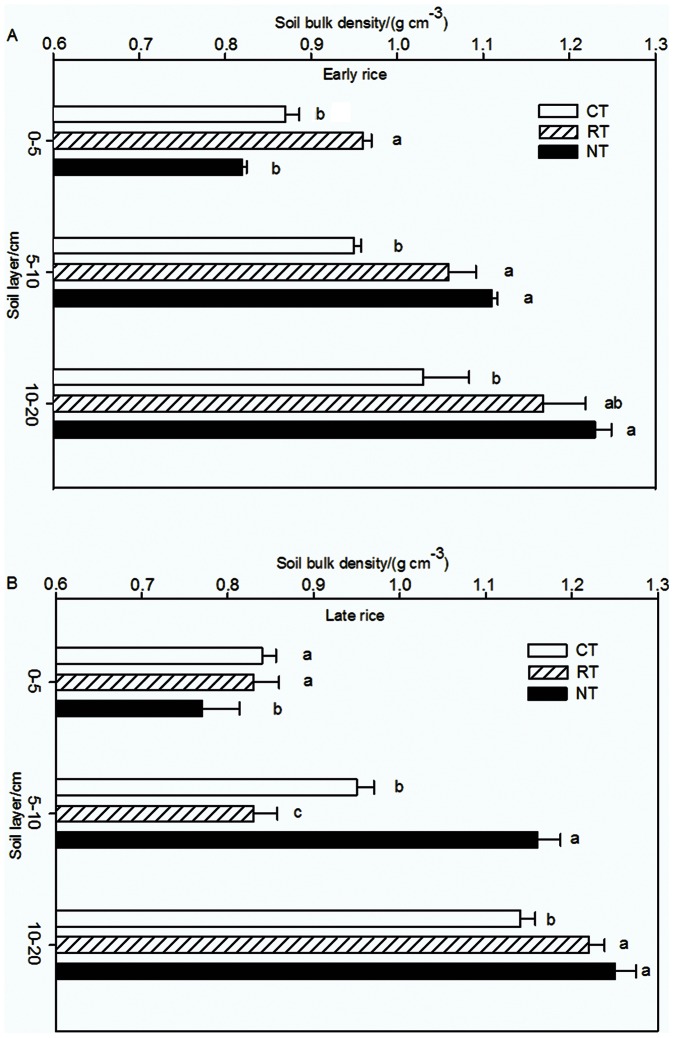
Soil bulk density of different tillage treatments in 2008 (A for the early rice season and B for the late rice season). Data are means of three replications; means followed by different letters are significantly different at *P<0.05.* Sampling was done during the harvest of the early and late rice in 2008.

Regardless of tillage practice, ρ_b_ increased with soil depth, but ρ_b_ increased more in NT than the other tillage treatments. Among the tillage treatments, ρ_b_ varied in the order of RT>CT>NT at 0–5 cm depth (Fig. 1), but varied in the order of NT>CT>RT at 5–10 cm depth for both the early and the late growing season. Compared with NT, ρ_b_ was lower at 5–10 cm and 10–20 cm depth under RT and CT. Figure 1 indicated that ρ_b_ under RT changed dramatically during the rice growing season, especially at 0–10 cm depth. At 0–5 cm and 5–10 cm depth, ρ_b_ under RT were higher in the early rice season than in the late rice season (0.23 vs. 0.13 g cm^−3^). In both the early and the late rice growing season, ρ_b_ under RT was significantly different from that under NT (Tukey HSD. early rice season: 0–5 cm, df = 8 F = 31.907 *P<0.05*; 5–10 cm, df = 8 F = 20.100 *P<0.05*; 10–20 cm, df = 8 F = 10.323 *P<0.05*. Late rice season: 0–5 cm, df = 8 F = 35.083 *P<0.05*; 5–10 cm, df = 8 F = 43.017; *P<0.05*; 10–20 cm df = 8 F = 8.089 *P<0.05*). Because of minimal soil disturbance, ρ_b_ under NT increased greatly in the deeper soil layers (Fig. 1). The significant change of ρ_b_ in RT may be due to soil disturbance and crop residue incorporation, whereas NT had the crop residue remaining on the soil surface.

### Soil Porosity

**Figure 2:**
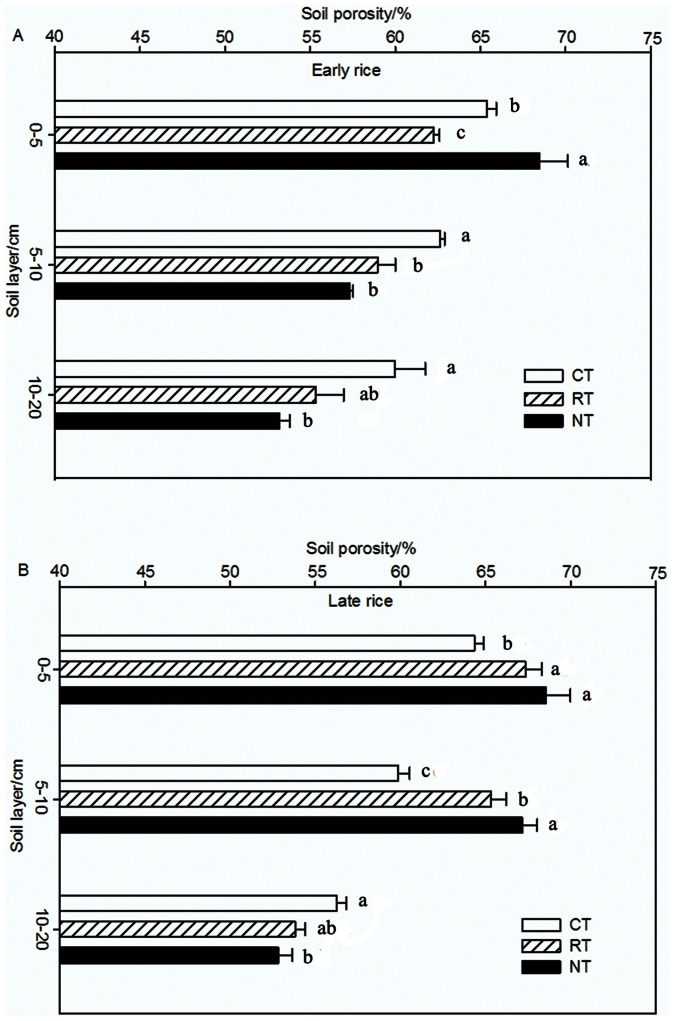
Soil porosity of different tillage treatments in 2008 (A for the early rice season and B for the late rice season). Data are means of three replications; means followed by different letters are significantly different at *P<0.05*.

Soil porosity decreased with soil depth among all the treatments (Fig. 2). For the early rice season, SP at 0−5 cm depth was 68.48%, 63.18% and 61.03% for NT, CT and RT, respectively. Tukey HSD statistical test showed that SP for NT and CT significantly differed with that of RT (0−5 cm, df = 8 F = 69.651 *P<0.05*; 5−10 cm, df = 8 F = 18.589 *P<0.05*; 10−20 cm, df = 8 F = 10.393 *P<0.05*). The order of SP at depths of 5−10 cm and 10−20 cm varied with CT>RT> NT; and SP for CT and RT were 11.5% and 8.9% higher than that of NT, respectively. The trend of SP in the late rice season varied similarly with that of the early rice season (0−5 cm, df = 8 F = 30.167 *P<0.05*; 5−10 cm, df = 8 F = 195.166 *P<0.05*; 10−20 cm df = 8 F = 6.957 *P<0.05*). Conversion of traditional tillage to NT, SP at 5−10 cm depth was higher 1.83% and 7.27% than that for CT and RT, respectively. Compared with NT, SP for CT significantly increased at 10−20 cm depth in the early rice season. During the early rice growing season, SP at 5−10 cm depth varied in the order of CT>RT>NT (*P*<0.05). However, during the late rice season, SP at 5−10 cm depth followed in the order of NT>RT>CT (*P*<0.05) and 9.84% and 6.35% higher for NT and RT than for CT, respectively.

### CH_4_ Emission

**Figure 3:**
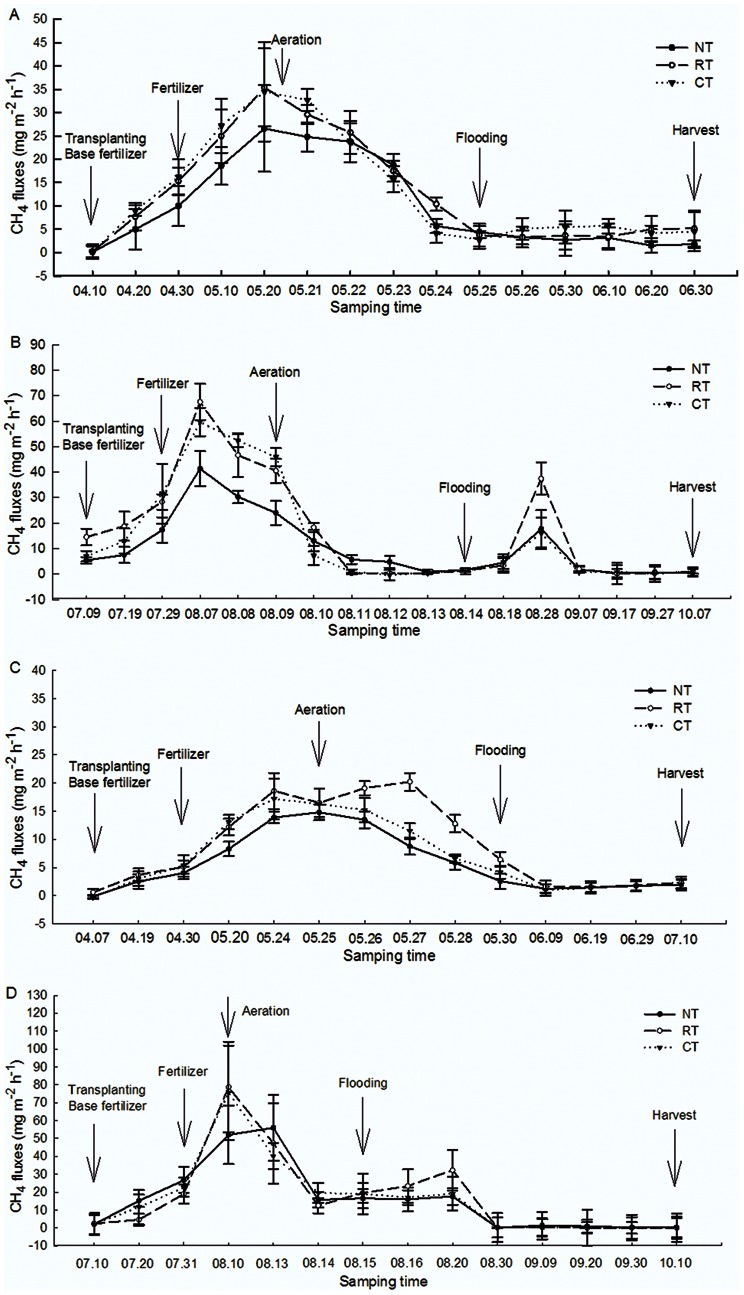
CH_4_ flux under different tillage during the rice growing seasons (A, B for the early rice season and the late rice season in 2007; C, D for the early rice season and the late rice season in 2008, respectively). Vertical bars represent standard errors of the mean (n = 3).The arrows in the figures indicate the time of field operation.

For the early rice season, paddy soil was the atmospheric source of CH_4_ under all treatments in both years. The flux of CH_4_ showed a single peak pattern characterized by three stages (Fig. 3−a, b). The first stage was the increasing stage of CH_4_ emission. The flux of CH_4_ showed a continuous increase under all the treatments and attained the highest fluxes during the aeration stage. The CH_4_ emissions from both CT and RT displayed similar trends and were higher than that from NT (Fig. 3−a, b). The second stage was the decreasing stage of CH_4_ emission. The flux of CH_4_ decreased rapidly from the aeration stage to the flooding stage during the early rice season. The emission fluxes in 2007 and 2008 were in the same order of RT>CT>NT and significant differences among the treatments were observed in 2008 (*P*<0.05). The third stage was characterized by stable CH_4_ emission. The flux of CH_4_ remained at a low level and tended to be stable from the flooding stage to the harvest stage. In 2008, the cumulative emissions were 228.3, 276.3 and 188.1 kg ha^−1^ for CT, RT and NT, respectively and were 17.9%, −1.7% and 16.2% lower in 2007, respectively. The difference between 2007 and 2008 was possibly due to weather differences.

The flux of CH_4_ for the late rice season (Fig. 3-a, b) showed a double emission peak. Before flooding, the CH_4_ emission flux exhibited similar trends to that of the early rice season. However, there was another small peak emission after the flooding stage which was lower than the first peak emission. For both years, CT had higher CH_4_ emission in the second peak fluxes than that of RT and NT. The cumulative emissions of CH_4_ for the late rice season in 2008 were 526.2, 565.5 and 506.2 kg ha^−1^ for CT, RT and NT, respectively; and 68.5%, 39.3% and 140.8% higher than in 2007, respectively.

The cumulative CH_4_ emission under NT was lower than that under CT and RT (Fig. 3-a, b), and the difference was significant at the peak emission (*P*<0.05). In contrast, CT emitted more CH_4_ during the early and the late rice growing seasons, with a higher peak emission than that of NT and RT (Fig. 3-a, b).

**Figure 4:**
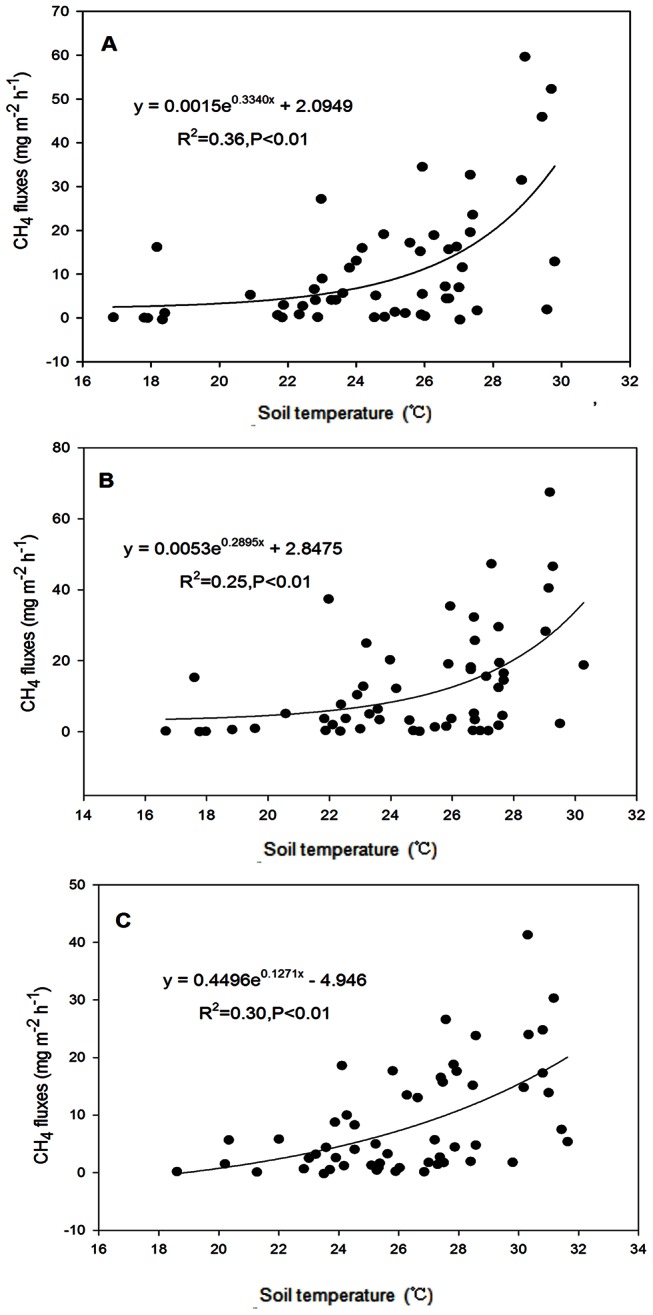
Relationship between soil temperature and CH_4_ emission from paddy fields (A for CT at 5 cm depth soil, B for RT at 5 cm depth soil, and C for NT at surface soil ). R^2^: coefficient of determination.

The emission of CH_4_ was greatly correlated with soil temperature (Fig. 4). There were significant correlations between CH_4_ emission and soil temperature among the treatments. There was a significant correlation between CH_4_ emission and soil temperature at 5 cm depth for CT and RT, while significant correlation for NT was at the soil surface.

Compared with the GWPs of CH_4_ emission (over 100 years), the mean value of 2007 and 2008 for NT was significantly lower than for CT and RT (*P*<0.05) with 16814, 18988 and 14112 kg ha^−1^ CO_2_-equivalent for NT, RT and CT, respectively.

### N_2_O Emission

**Figure 5:**
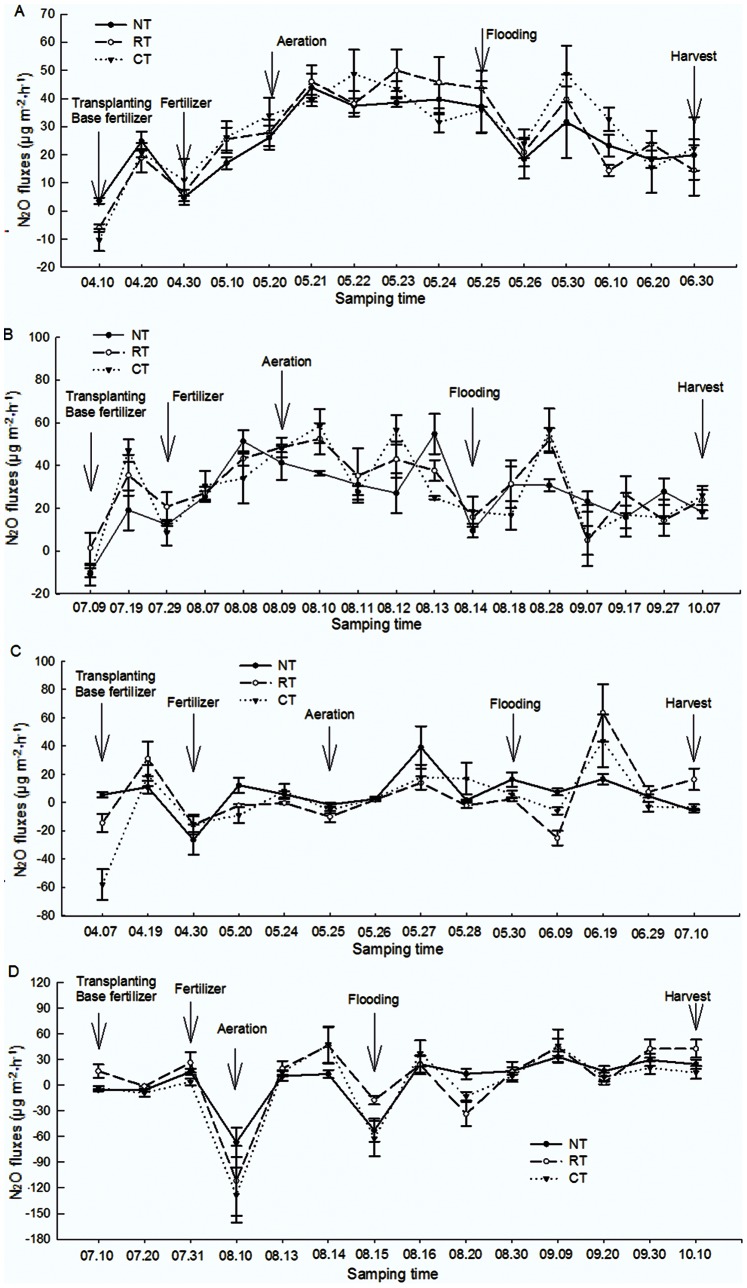
N_2_O flux under different tillage during the rice growing seasons (A, B for the early rice season and the late rice season in 2007; C, D for the early rice season and the late rice season in 2008, respectively). Vertical bars represent standard errors of the mean (n = 3).The arrows in the figures indicate the time of field operations.

The N_2_O emission exhibited an impulse type for both the early and the late rice season in 2007 and 2008 (Fig. 5-a, b). Regardless of tillage methods, the N_2_O emission exhibited an emission peak after tillage, aeration and flooding. The first peak of N_2_O emission appeared ∼10 days after tillage. The emission varied in the order of RT>CT>NT in 2008, and RT was significantly higher than NT (*P*<0.05). The emission order was NT>CT>RT in 2007, but no significant differences among treatments (*P*<0.05) were observed. The N_2_O emission fluxes decreased after fertilizer application, but aeration and flooding triggered emission peaks.

All the three tillage treatments were weak sources of N_2_O (Table 3). In 2008, the cumulative N_2_O emission was 0.01, 0.30 and 0.30 kg ha^−1^ for CT, RT and NT, respectively. However, the emissions in 2008 were nearly 60% lower than those in 2007 for all the treatments. The annual difference of the cumulative emission was possibly due to influences from meteorological factors (i.e., temperature, precipitation). Regardless of the year, the N_2_O emission fluxes for NT was more stable than that for CT and RT, ranging from 13.1−33.0 µg m^−2^ h^−1^ in the late rice season. On the other hand, the emission fluxes for RT and CT changed greatly from day to day. However, aeration strongly influenced N_2_O emissions for all the treatments. In general, about 68%−81% of the cumulative N_2_O emissions occurred from aeration to harvest in 2007. Compared with CT and RT, NT significantly increased the N_2_O emission from aeration to harvest in both 2007 and 2008 (*P*<0.05).

**Table 3:** Cumulative N_2_O emissions of each farm operation phase during the rice growing period.

Year			Treatments		
			CT (kg ha^−1^)	RT (kg ha^−1^)	NT (kg ha^−1^)
2007	Early rice	Before aeration	0.09b	0.08c	0.10a
		During aeration	0.13a	0.12a	0.10b
		After aeration	0.24a	0.19b	0.19b
	Late rice	Before aeration	0.12b	0.13a	0.06c
		During aeration	0.10b	0.11a	0.10b
		After aeration	0.16c	0.18b	0.18a
Total emission			0.84a	0.82b	0.72c
2008	Early rice	Before aeration	−0.11c	0a	−0.03b
		During aeration	0b	0b	0.02a
		After aeration	0.09b	0.13a	0.09b
	Late rice	Before aeration	−0.16b	−0.07a	−0.05a
		During aeration	−0.04c	−0.02a	−0.03b
		After aeration	0.22c	0.26b	0.29a
Total emission			0.01c	0.30a	0.30b

Values are means of three replications for each treatment; means followed by different letters are significantly different at *P<0.05*.

Compared with the GWPs of N_2_O emission (over 100 years), the mean value of 2007 and 2008 for CT was significantly lower than that for RT and NT (*P*<0.05). The values were 126.7, 166.9 and 152.0 kg ha^−1^ CO_2_-equivalent for NT, RT and CT, respectively.

## Discussion

### CH_4_ Emission

Large variations in CH_4_ emission were observed during the rice growing seasons, which may be attributed to differences in meteorological conditions. However, soil tillage had significant effects on CH_4_ emission across the entire rice growing seasons. In this study, NT had a lower CH_4_ emission compared with other treatments (*P*<0.05), which is consistent with the results of Zhang et al. [36]. Gregorich et al. attributed the differences in gas fluxes between NT and CT to differences in the physical environment [37]. Wang et al. indicated that the major differences in CH_4_ production zone resulted from the disturbed depth by the different tillage methods [38]. Therefore, the CH_4_ production zone may vary according to the adopted tillage method. Wang et al. also reported that the main oxidation zone of CH_4_ was the root surface and the interface between soil and water [38]. The rice residues retention may have increased the soil oxide layer. In this study, NT significantly increased the SP at 0−5 cm depth (Fig. 2), and thus had a larger oxide layer than other treatments, which may be beneficial to the oxidization of CH_4_. Regina et al. indicated that CH_4_ oxidation rate was higher when there were more macro-pores or fewer micro-pores in the soil [39]. In addition, CH_4_ emission was influenced by soil temperature and soil redox potential (Eh). Yu et al. [40] reported that CH_4_ emission showed an exponential decrease by an Eh increase.

In this study, the crop residues were distributed on the soil surface under NT. Furthermore, the decomposition of residues consumed limited soil dissolved oxygen. All these factors discussed above resulted in Eh decrease and consequently a reduction of CH_4_ emission under NT. Khalil et al. [41] observed an increase in CH_4_ emissions from paddy fields with increasing soil temperature. In this study, temperature was another major factor affecting CH_4_ emission (Fig. 4). In general, NT decreased soil temperature especially during the hotter days. Therefore, low temperatures also reduced the CH_4_ emission when compared with other treatments.

In this study, CH_4_ emission from the late rice season was 65% higher than that from the early rice season, which indicates that the late rice paddy is the principal CH_4_ source in double paddy fields. Temperature was the major reason for the differences in the CH_4_ emission pattern between the early and the late rice season. The soil temperature had a predictive functional relationship with CH_4_ emission. Zhu et al. [42] and Bossio et al. [43] reported a strong correlation between CH_4_ emission and soil temperature. Furthermore, Whalen and Reeburgh [44] reported that temperature had important influence on CH_4_ emission from soils and the combination of high soil moisture and low temperature was favorable to decrease CH_4_ emission. In this study, an exponential model was used for fitting CH_4_ emission and soil temperature. Our results showed that there was a significant correlation between CH_4_ emission and soil temperature. But the coefficient of determination was not high, and this may be due to the fluctuation of soil temperature influenced by the alternation of wetting and drying in paddy. In this experimental area, the late rice season was the hottest time of the summer. Therefore, high temperatures enhanced the decomposition rate of crop residues in the moist environment. During the decomposition process of crop residues, a large number of organic compounds are produced and oxygen is consumed, thus decreasing the soil Eh, leading to an increase in the possibility of CH_4_ emission. In contrast to the warm temperatures of the late rice season, the air temperatures of the early rice season were lower, which resulted in slower crop residue decomposition and therefore little CH_4_-substrate. Hence, these differences in weather factors (e.g., temperature) resulted in the different characteristics of CH_4_ between the early and the late rice seasons.

### N_2_O Emission

In our study, the fluxes of N_2_O emission show a great fluctuation during the rice growth seasons, but it remained at a low level. Indeed, the N_2_O emission was strongly influenced by external factors and many emission peaks occurred during the rice growing season. The emission of N_2_O was dramatically different between the two years. This difference is possibly due to the variations in weather. Some studies show that extreme precipitation and drying could increase N_2_O emission [45], [46]. Hao et al. [47] reported that aeration and water flooding led to outbreaks of emissions. The precipitation in 2007 was much higher than the precipitation in 2008. This precipitation difference may explain the fluctuations of N_2_O emissions between the two years.

The N_2_O emission differences among the treatments were possibly due to farm operations (e.g., tillage, drainage). Some results indicated that N_2_O production and emission was greatly influenced by tillage because of the breaking of the soil uniformity [48]. Nitrogen (mainly as NO_3_^−^-N or NH_4_^+^-N) can remain stable in homogeneous soil and thus may decrease N_2_O production. Tillage practices change the soil nutrients and crop residue distribution. The distribution of soil nutrients was relatively even under CT and RT by cutting, mixing, overturning the soil and crop residues. However, the crop residues were well-distributed only in the 0–8 cm soil layer under RT because of the shallow tilled depth. High stratification ratio of soil nutrients (e.g., N, SOC) across different depths is observed in NT systems [48], [49], which means that the soil nutrient distributions are not even among different depths. Therefore, the different distribution of crop residues and soil nutrients among the treatments influences the N_2_O production and emission. In addition, similar to CH_4_, N_2_O emission is also influenced by soil Eh. Weier et al. reported that the rate of N_2_O emission decreased with increasing soil reducibility [49]. Generally, crop residues in CT are mainly distributed within the plow layer (0–20 cm) and had a strong redox potential due to decomposition of crop residues. Therefore, N_2_O produced from CT soils tended to be further deoxidized to N_2_, which consequently decreased N_2_O emission. Similar results were also reported by Steinbach and Alvarez [50] who observed NT increased N_2_O emission.

### Conclusion

Paddy fields with rice residues retention were a source of atmospheric CH_4_, regardless of the tillage practice. Compared with other treatments, NT reduced CH_4_ emission among the rice growing seasons. The GWPs (based on CH_4_ emission) under NT was significantly *(P<0.05*) lower than that of CT and RT. The N_2_O emission was vulnerable to external influences and varied greatly during the rice growing seasons. Although the cumulative emission under NT was more than other treatments, GWPs of N_2_O was relative low compared to that of CH_4_. Therefore, N_2_O emission was a weak source of GHG in paddy fields. The GWPs (based on CH_4_ and N_2_O) of NT is lower than that of CT and RT. Thus, adoption of NT is beneficial in GHG mitigation and could be a good practice of carbon-smart agriculture in double rice cropped regions.
